# About the Definition of the Local Equilibrium Lattice Temperature in Suspended Monolayer Graphene

**DOI:** 10.3390/e23070873

**Published:** 2021-07-08

**Authors:** Marco Coco, Giovanni Mascali, Vittorio Romano

**Affiliations:** 1 Department of Industrial Engineering and Mathematical Sciences, Università Politecnica delle Marche, Via Brecce Bianche, 12, 60131 Ancona, Italy; m.coco@univpm.it; 2 Department of Mathematics and Computer Science, Università degli Studi della Calabria and INFN-Gruppo c. Cosenza, 87036 Cosenza, Italy; 3 Department of Mathematics and Computer Science, Università degli Studi di Catania, Viale Andrea Doria 6, 95125 Catania, Italy; romano@dmi.unict.it

**Keywords:** local equilibrium temperature, electron–phonon transport, graphene, macroscopic models, maximum entropy principle

## Abstract

The definition of temperature in non-equilibrium situations is among the most controversial questions in thermodynamics and statistical physics. In this paper, by considering two numerical experiments simulating charge and phonon transport in graphene, two different definitions of local lattice temperature are investigated: one based on the properties of the phonon–phonon collision operator, and the other based on energy Lagrange multipliers. The results indicate that the first one can be interpreted as a measure of how fast the system is trying to approach the local equilibrium, while the second one as the local equilibrium lattice temperature. We also provide the explicit expression of the macroscopic entropy density for the system of phonons, by which we theoretically explain the approach of the system toward equilibrium and characterize the nature of the equilibria, in the spatially homogeneous case.

## 1. Introduction

The definition of temperature in non-equilibrium situations is among the most controversial questions in thermodynamics and statistical physics (for a comprehensive review, the interested reader is referred to [1,2,3,4]). Several approaches have been adopted, trying to generalize what is already known in thermostatics, e.g., the non-equilibrium temperature is defined as the partial derivative of the entropy with respect to the internal energy, even if this translates the problem to the formulation of non-equilibrium entropy, which can be introduced on the basis of considerations of extended thermodynamics or non-equilibrium statistical mechanics. In [5], a contact temperature is introduced in which a non-equilibrium system is put in contact with a control equilibrium environment and the global heat flux through the boundaries is zero, the contact temperature being that of the environment. In [6], by extending Rugh’s ideas [7] to non-equilibrium situations, a configurational temperature is introduced, related to the forces between the particles of the system under consideration.

The attempt to validate an approach by comparison with experiments is intrinsically ambiguous since it is not well understood as to what it is indeed measured by the apparatus.

Here, we focus on the issue of the definition of the local equilibrium temperature in the case of the lattice temperature of graphene. Recently, this material has received much attention, in particular because of its thermal and electrical properties [8]. At the kinetic level, the thermal effects are described with a phonon gas, which obeys a suitable Boltzmann equation. Likewise, charge transport is accurately described by a semiclassical transport equation. Electron–phonon scatterings couple the two subsystems. In [9,10] electro-thermal effects were analyzed by a Monte Carlo simulation (for results based on a finite difference scheme, see [11]; for results based on a discontinuous Galerkin scheme, see [12]), adapting the method in [13,14,15] with a description of the phonon–phonon scatterings based on a Bhatnagar–Gross–Krook (BGK) approximation, where a proper definition of a local temperature of the graphene crystal lattice is needed. This latter is deduced from the conservation properties of the phonon–phonon collision terms.

A different approach for the analysis of the electro-thermal effects in graphene was adopted in [16,17], where a hydrodynamical model based on the moment method with closure relations obtained by exploiting the maximum entropy principle (MEP) [18,19,20,21], was formulated (for similar results with phonons considered to be a thermal bath, see [22,23]). Here, in agreement with rational extended thermodynamics [24], a definition of a different local temperature was introduced, which is directly related to the Lagrange multiplier associated to the phonon energy (a hybrid MC–hydrodynamical approach can be found in [25]). We remark that the problem is indeed very complex because, even if we restrict the analysis to the phonons, there are several degrees of freedom represented by the different phonon branches, each of them having its own temperature. The phonon–phonon collision operator creates a mechanism of energy exchange, which pushes the several degrees of freedom to a common temperature in the absence of an external field. Therefore, there are two important questions to face: to give, out of equilibrium, a suitable definition of temperature both for each phonon species and for the crystal lattice.

On account of the difficulties with the experimental results, we face the question of analyzing the meaning of the above-mentioned two local lattice temperatures with two numerical experiments. In the first one, coupled electron–phonon simulations are performed, while in the second one, only phonon transport is considered. The hydrodynamic model in [17] is adopted, with a more accurate approximation of the phonon–phonon collision operator. The obtained results, particularly the second set of simulations, strongly indicate that the appropriate definition of the local equilibrium lattice temperature should be that based on the energy Lagrange multipliers, also in accordance with the current non-equilibrium theory [24,26]. In fact, in the second numerical experiment, it remains constant, as it should be, when the total energy is conserved at variance with the other local temperature, which, instead, presents an overshoot during the transient before asymptotically tending to the other temperature. Nevertheless, the definition based on the conservation property of the phonon collision operator has also a physical meaning and can be regarded as a measure of how fast the system is trying to approach the equilibrium.

The plan of the paper is the following. In Section 2, the kinetic model and the definition of the first local temperature are presented. In Section 3, a macroscopic model and the definition of the second local temperature are introduced. In Section 4, the results of the numerical simulations are shown and the difference between the two local temperatures is critically highlighted. Eventually, in Section 5, we furnish the expression for the macroscopic entropy density for the phonon system by which we prove the asymptotic stability of the phonon equilibrium states and theoretically justify the behavior of the phonon temperatures in the second numerical experiment.

## 2. The Kinetic Model and the Definition of the First Local Temperature

Graphene consists of carbon atoms arranged in a honeycomb hexagonal lattice. The charge transport is essentially due to the electrons, which are located around the *Dirac points*, *K* and $K^{\prime }$, which are the vertices of the hexagonal primitive cell of the reciprocal lattice. At the Dirac points, the valence and conduction bands touch each other, which makes graphene a gapless semimetal. Moreover, having the energy bands in an approximately conical shape, electrons behave as massless Dirac fermions [8]. If high enough, Fermi levels are considered. It is possible to neglect the dynamics of the electrons in the valence bands, being that the latter ones are fully occupied in this case [27]. Such a situation is similar to n-type doping for traditional semiconductors. As said, around the equivalent Dirac points, the band energy $\varepsilon_{\ell }$ is approximately linear.

(1) $$\varepsilon_{\ell }=\;\hbar \;v_{F}\left|k-k_{\ell }\right|,$$

and the group velocity is given by the following:

$$v_{\ell }=\frac{1}{\hbar }\;\nabla_{k}\;\varepsilon_{\ell }=v_{F}\frac{k-k_{\ell }}{|k-k_{\ell }|}\;.$$

where k is the electron wave-vector, $v_{F}$ is the (constant) Fermi velocity, *ℏ* is the reduced Planck constant, and $k_{\ell }$ is the position of the Dirac point $\ell =K,K^{\prime }$.

In the framework of the semiclassical kinetic theory, relative to the electrons in the conduction band, the charge transport is described by two Boltzmann equations for the *K* and $K^{\prime }$ valleys:

(2) $$\begin{array}{c} \left.\frac{\partial f_{\ell }\left(t,x,k\right)}{\partial t}+v_{\ell }\cdot \nabla_{x}f_{\ell }\left(t,x,k\right)\right.{\left.-\frac{e}{\hbar }E\cdot \nabla_{k}f_{\ell }\left(t,x,k\right)=\frac{df_{\ell }}{dt}\left(t,x,k\right)\right|}_{e-ph}, \end{array}$$

where $f_{\ell }\left(t,x,k\right)$ represents the distribution function of the electrons in the valley *ℓ* (*K* or $K^{\prime }$), at position x, time *t*, and with wave-vector k. By $\nabla_{x}$ and $\nabla_{k}$, we denote the gradients with respect to the position and the wave-vector, respectively; *e* is the elementary (positive) charge; and E is the external applied electric field.

The collision term at the right-hand side of (2) describes the scatterings occurring between electrons and phonons. They can be with longitudinal, transversal, acoustic or optical phonons, which are labeled by LA, TA, LO and TO, respectively. Both the acoustic and the optical phonon scatterings are intra-valley and intra-band. One also has to take into account the electron scattering with *K* phonons, which is inter-valley, pushing electrons from a valley to the nearby one. The general form of the collision term is as follows:

$$\begin{array}{ccc} {\left.\frac{df_{\ell }}{dt}\left(t,x,k\right)\right|}_{e-ph}=\sum_{\ell^{\prime }}\left[\int_{I_{\ell^{\prime }}}S_{\ell^{\prime },\ell }\left(k^{\prime },k\right)\;f_{\ell^{\prime }}\left(t,x,k^{\prime }\right)\left(1-f_{\ell }\left(t,x,k\right)\right)dk^{\prime }\right. & & \\ \left.-\int_{I_{\ell }}S_{\ell ,\ell^{\prime }}\left(k,k^{\prime }\right)\;f_{\ell }\left(t,x,k\right)\left(1-f_{\ell^{\prime }}\left(t,x,k^{\prime }\right)\right)dk^{\prime }\right], & \end{array}$$

where the total transition rate $S_{\ell^{\prime },\ell }\left(k^{\prime },k\right)$ is given by the sum of the contributions of the several types of scatterings [17]:

(3) $$\begin{array}{ccc} S_{\ell^{\prime },\ell }\left(k^{\prime },k\right)\; & = & \;\sum_{\mu }{\left|G_{\ell^{\prime },\ell }^{\left(\mu \right)}\left(k^{\prime },k\right)\right|}^{2}\;\left[\left(g_{\mu }^{-}+1\right)\delta \;\left(\varepsilon_{\ell }\left(k\right)-\varepsilon_{\ell^{\prime }}\left(k^{\prime }\right)+\hbar \;\omega_{\mu }\right)\right.\\ & & \left.+g_{\mu }^{+}\;\delta \left(\varepsilon_{\ell }\left(k\right)-\varepsilon_{\ell^{\prime }}\left(k^{\prime }\right)-\hbar \;\omega_{\mu }\right)\right]. \end{array}$$

The index μ labels the μth phonon mode and $\omega_{\mu }$ is its angular frequency; $I_{\ell }$ labels the region in the Brillouin zone corresponding to the valley *ℓ*. The ${\left|G_{\ell^{\prime },\ell }^{\left(\mu \right)}\left(k^{\prime },k\right)\right|}^{2}$’s are the electron–phonon coupling matrix elements, which describe the interaction mechanism of a μth phonon with an electron, going from the state of wave-vector $k^{\prime }$ belonging to the valley $\ell^{\prime }$ to the state with wave-vector k, belonging to the valley *ℓ*. The symbol δ denotes the Dirac distribution; $g_{\mu }\left(t,x,q\right)$ is the phonon distribution for the μ-type phonons; and q is the phonon wave-vector belonging to the first Brillouin zone B, measured from Γ or *K*, respectively, for Γ and *K* phonons. In Equation (3), $g_{\mu }^{\pm }=g_{\mu }\left(q^{\pm }\right)$, where $q^{\pm }=\pm \left(k^{\prime }-k\right)$, stemming from the momentum conservation. The *K* and $K^{\prime }$ valleys can be treated as equivalent; therefore, in the following, we will take into account a unique electron population.

Similarly, the evolution of the phonon populations is determined by the following Boltzmann equations for the phonon distributions as follows:

(4) $$\begin{array}{ccc} & & \frac{\partial g_{op}}{\partial t}=C_{op},\;op=\mathrm{LO},\mathrm{TO},\mathrm{ZO},K, \end{array}$$

(5) $$\begin{array}{ccc} & & \frac{\partial g_{ac}}{\partial t}+c_{ac}\cdot \nabla_{x}g_{ac}=C_{ac},\;ac=\mathrm{LA},\mathrm{TA},\mathrm{ZA}, \end{array}$$

where $c_{ac}=\nabla_{q}$$\omega_{ac}$ is the acoustic phonon group velocity.

The optical phonon group velocity can be neglected because of the Einstein approximation $\epsilon_{op}=\hbar \omega_{op}\approx const$, which can be used for their dispersion relation, while, regarding the LA and TA phonons, the Debye approximation can be employed: $\omega_{ac}=\epsilon_{ac}/\hbar =c_{ac}\left|q\right|$, with $c_{ac}$ the sound speed of the branch *ac* = LA, TA. Eventually, the dispersion relation of the ZA phonons is approximately quadratic: $\epsilon_{\mathrm{ZA}}=\hbar \alpha_{\mathrm{ZA}}{\left|q\right|}^{2}$, with $\alpha_{ZA}$= 0.62 nm${}^{2}$/ps.

The phonon collision term splits into two parts as follows:

(6) $$\begin{array}{ccc} & & C_{\mu }=C_{\mu }^{p-e}+C_{\mu }^{p-p},\;\mu =\mathrm{LA},\mathrm{TA},\mathrm{ZA},\mathrm{LO},\mathrm{TO},\mathrm{ZO},K. \end{array}$$

where the term $C_{\mu }^{p-e}$ is the phonon–electron collision operator, while $\;C_{\mu }^{p-p}$ represents the phonon–phonon interactions, which are very difficult to treat from a numerical point of view. For this reason, a BGK approximation is commonly used for them [16]:

$$C_{\mu }^{p-p}=-\frac{g_{\mu }-g_{\mu }^{LE}}{\tau_{\mu }},$$

which describes the relaxation of each phonon branch toward the equilibrium condition that corresponds to the local equilibrium Bose–Einstein distributions.

(7) $$\begin{array}{ccc} & & g_{\mu }^{LE}={\left[e^{\hbar \omega_{\mu }/k_{B}T_{L}}-1\right]}^{-1}. \end{array}$$

**Figure 1:**
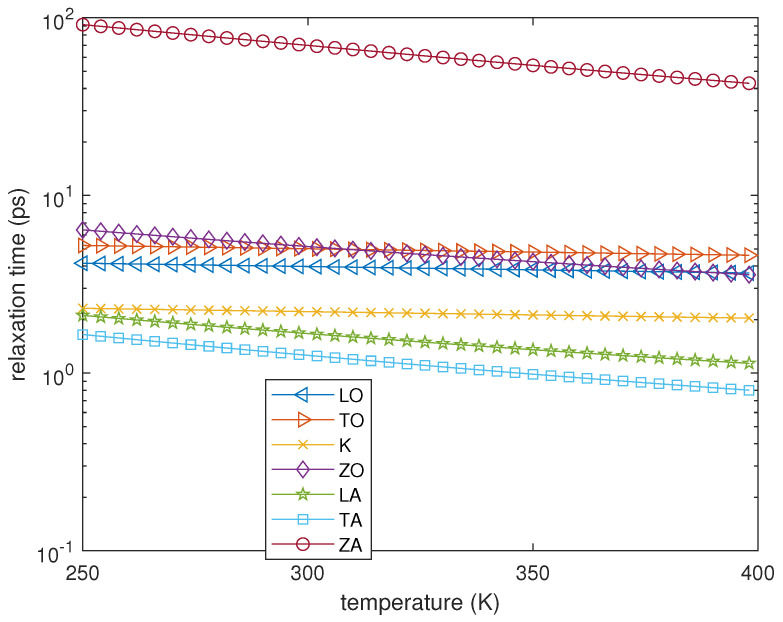
Relaxation times versus the local temperature.

The functions $\tau_{\mu }=\tau_{\mu }\left(T_{\mu }\right)$, which are reported in Figure 1, are the relaxation times, each of them depending only on the temperature $T_{\mu }$ of the branch under consideration. The temperature $T_{L}$ is the same for each phonon population and is defined as follows.

If the phonon distributions $g_{\mu }$ are known, the average phonon energy densities can be calculated as follows:

(8) $$\begin{array}{ccc} & & W_{\mu }=\frac{1}{{\left(2\pi \right)}^{2}}\int_{B}\hbar \omega_{\mu }\;g_{\mu }\;dq, \end{array}$$

where $\frac{1}{{\left(2\pi \right)}^{2}}$ represents the μth phonon density of the states, and the temperature $T_{\mu }$ of each phonon branch is determined from the following condition:

(9) $$\int_{B}\hbar \omega_{\mu }g_{\mu }\left(q\right)dq=\int_{B}\hbar \omega_{\mu }\;{\left[e^{\hbar \omega_{\mu }/k_{B}T_{\mu }}-1\right]}^{-1}dq,$$

where μ varies over the phonon branches.

From the conservation of the total phonon energy, under processes involving only phonons, the following relation has to hold:

(10) $$\begin{array}{c} \sum_{\mu }\frac{W_{\mu }-W_{\mu }^{LE}}{\tau_{\mu }}=0 \end{array}$$

where $W_{\mu }^{LE}$ is calculated by means of (7) and (8). The above-written condition allows one to state the following definition of local temperature.

**Definition** **1.** *We define $T_{L}$ as the solution of the non-linear relation (10), which can be obtained numerically; see e.g., [28,29] where this local temperature is called scattered phonon pseudo-temperature.*

It is possible to prove that (10) admits a unique solution. For further details, we refer to [10], where the previous approach was adopted to devise a simulation scheme for the electron–phonon transport in graphene.

## 3. A Macroscopic Model and the Definition of the Second Local Temperature

Macroscopic models can be derived from the kinetic one [17,19,30] by taking suitable moments of the distribution functions as state variables. Here, we present in some detail only the evolution equations of the phonon variables, and refer the interested reader to [17] for a complete treatment of the problem.

If one chooses a certain number of moments of the electron and phonon distributions as state variables, the extra fluxes and the production terms, which are present in the corresponding balance equations, are additional unknown quantities, which require constitutive relations in terms of the state variables. By exploiting the maximum entropy principle (MEP), the electron and phonon distributions can be estimated by the so-called maximum entropy distributions $f_{MEP}$ and $g_{\mu ,MEP}$, μ = LO, TO, ZO, K, LA, TA, ZA, which solve the following maximization problem:

$$\max_{f,g_{\mu }}S\left[f,g_{\mu }\right],$$

under the constraint that the moments chosen as fundamental variables are known. $S[f,g_{\mu }]$ is the total entropy of the physical system, which depends on the electron and phonon distribution functions *f* and $g_{\mu }$, and whose expression is reported in [17].

In particular, for the phonons, the following moments can be chosen:

(11) $$\begin{array}{ccc} & & W_{op}=\frac{1}{{\left(2\pi \right)}^{2}}\int_{B}\hbar \omega_{op}g_{op}\;dq,\;P_{op}=\frac{1}{{\left(2\pi \right)}^{2}}\int_{B}\hbar qg_{op}\;dq,\;op=\mathrm{LO},\mathrm{TO},\mathrm{ZO},K,\; \end{array}$$

(12) $$\begin{array}{ccc} & & W_{ac}=\frac{1}{{\left(2\pi \right)}^{2}}\int_{R^{2}}\hbar \omega_{ac}g_{ac}\;dq,\;Q_{ac}=\frac{1}{{\left(2\pi \right)}^{2}}\int_{R^{2}}\hbar \omega_{ac}c_{ac}g_{ac}\;dq,\;ac=\mathrm{LA},\mathrm{TA},\mathrm{ZA},\; \end{array}$$

which respectively represent the energy and momentum densities of the optical phonons and the energy and energy flux densities of the acoustical phonons. Solving the above constrained maximization problem, one obtains the following:

$$\begin{array}{ccc} g_{op,MEP} & = & \frac{1}{\exp\left(\lambda_{W_{op}}\epsilon_{op}+\hbar \;q\cdot \lambda_{P_{op}}\right)-1},\;op=\mathrm{LO},\mathrm{TO},\mathrm{ZO},K,\\ g_{ac,MEP} & = & \frac{1}{\exp\left(\lambda_{W_{ac}}\epsilon_{ac}+\epsilon_{ac}\;c_{ac}\cdot \lambda_{Q_{ac}}\right)-1},\;ac=\mathrm{LA},\mathrm{TA},\mathrm{ZA}, \end{array}$$

where the λs are the Lagrange multipliers arising from the presence of the constraints.

In order to tackle the problem of the inversion of the constraints, the distribution functions are linearized around their isotropic part, obtaining the following:

$$\begin{array}{ccc} g_{op,MEP} & \approx & \;\frac{1}{e^{\lambda_{W_{op}}\epsilon_{op}}-1}\left[1-\frac{e^{\lambda_{W_{op}}\epsilon_{op}}}{e^{\lambda_{W_{op}}\epsilon_{op}}-1}\;\hbar q\cdot \lambda_{P_{op}}\right],\;\;op=\mathrm{LO},\mathrm{TO},\mathrm{ZO},K,\\ g_{ac,MEP} & \approx & \;\frac{1}{e^{\lambda_{W_{ac}}\epsilon_{ac}}-1}\left[1-\frac{e^{\lambda_{W_{ac}}\epsilon_{ac}}}{e^{\lambda_{W_{ac}}\epsilon_{ac}}-1}\;\epsilon_{ac}\;c_{ac}\cdot \lambda_{Q_{ac}}\right],\;ac=\mathrm{LA},\mathrm{TA},\mathrm{ZA}. \end{array}$$

By substituting the latter expressions into the constraints (11) and (12) and by solving them with respect to the Lagrange multipliers, one finds the following:

(13) $$\begin{array}{ccc} & & \lambda_{W_{op}}=\frac{1}{\epsilon_{op}}\ln\left(1+\frac{yA\epsilon_{op}}{W_{o}p}\right),\;op=\mathrm{LO},\mathrm{TO},\mathrm{ZO},K,\; \end{array}$$

(14) $$\begin{array}{ccc} & & \lambda_{W_{ac}}\;\;\;=\;\;\;{\left(\frac{4\pi y\zeta (3)}{\hbar^{2}c_{ac}^{2}}\right)}^{\frac{1}{3}}W_{ac}^{-\frac{1}{3}},\;\;ac=\mathrm{LA},\mathrm{TA},\;\;\lambda_{W_{\mathrm{ZA}}}=\sqrt{\frac{\pi }{24\hbar \alpha_{\mathrm{ZA}}\;W_{\mathrm{ZA}}}}, \end{array}$$

(15) $$\begin{array}{ccc} & & \lambda_{P_{op}}=-\frac{A^{2}\epsilon_{op}^{2}}{4\hbar^{2}A_{1}}\frac{y}{W_{op}\left(W_{op}+Ay\epsilon_{op}\right)}P_{op},\;op=\mathrm{LO},\mathrm{TO},\mathrm{ZO},K, \end{array}$$

(16) $$\begin{array}{ccc} & & \lambda_{Q_{ac}}=-\frac{2}{3}{\left(\frac{4\pi y\zeta (3)}{\hbar^{2}c_{ac}^{8}}W_{ac}^{-4}\right)}^{\frac{1}{3}}Q_{ac},\;ac=\mathrm{LA},\mathrm{TA},\; \end{array}$$

(17) $$\begin{array}{ccc} & & \lambda_{Q_{\mathrm{ZA}}}=-\frac{16}{15}\frac{\sqrt{\pi }}{\alpha_{\mathrm{ZA}}^{3}\zeta \left(5/2\right)}{\left(\frac{24\hbar \alpha_{\mathrm{ZA}}}{\pi \;W_{\mathrm{ZA}}}\right)}^{\frac{7}{4}}Q_{\mathrm{ZA}},\; \end{array}$$

where $y=\frac{1}{{\left(2\pi \right)}^{2}}$, ζ(·) is the zeta function, $A=\frac{8\sqrt{3}}{9}\frac{\pi^{2}}{a_{0}^{2}},$$A_{1}=\frac{20\sqrt{3}}{729}\frac{\pi^{4}}{a_{0}^{4}},$ with $a_{0}=0.142\;$ nm being the nearest neighbor distance between the atoms in graphene.

Eventually, using the MEP distribution functions and the relations expressing the Lagrange multipliers as functions of the fundamental variables, it is possible to close the moment equations as follows:

(18) $$\begin{array}{ccc} \frac{\partial W_{op}}{\partial t} & = & C_{W_{op}},\;\frac{\partial P_{op}}{\partial t}=C_{P_{op}},\;\;op=\mathrm{LO},\mathrm{TO},K,\mathrm{ZO},\; \end{array}$$

(19) $$\begin{array}{ccc} \frac{\partial W_{ac}}{\partial t}+\nabla_{x}\cdot Q_{ac} & = & C_{W_{ac}},\;\frac{\partial Q_{ac}}{\partial t}+\nabla_{x}\cdot T_{ac}=C_{Q_{ac}},\;\;ac=\mathrm{LA},\mathrm{TA},\mathrm{ZA}, \end{array}$$

by means of the following closure relations for the production terms $C_{W_{op}},C_{P_{op}},C_{W_{ac}}$, $C_{Q_{ac}}$, and the acoustical phonon fluxes of energy fluxes $T_{ac}:=y\int_{B}\epsilon_{ac}c_{ac}⊗c_{ac}g_{ac}dq$:

(20) $$\begin{array}{ccc} & & C_{W_{\mu }}=-\frac{W_{\mu }-W_{\mu }^{LE}}{\tau_{\mu }},\;C_{P_{op}}=-P_{op}/\tau_{op},\; \end{array}$$

(21) $$\begin{array}{ccc} & & C_{Q_{ac}}=-Q_{ac}/\tau_{ac},\; \end{array}$$

(22) $$\begin{array}{ccc} & T_{LA/TA} & =\frac{c_{LA/TA}}{2}W_{LA/ZA}I,\;T_{ZA}=\frac{\zeta (3)}{\pi \hbar^{2}}{\left(\frac{\pi }{24\hbar \alpha_{ZA}}\;W_{ZA}\right)}^{\frac{3}{2}}I, \end{array}$$

where I is the identity matrix, the indices vary over the above-specified phonon branches, and only the productions due to the phonon interactions among themselves are considered.

In the previous section, we introduced a local lattice temperature by the relation (10), which stems from the properties of the phonon–phonon collision operator. However, the concept of temperature out of equilibrium is a subtle topic and still a matter of debate [24,26]. The rationale of the previous definition is that the collision operator *pushes* the system, in a characteristic time-related manner to the relaxation times toward a local equilibrium state with a single temperature for all the phonons. However, in statistical mechanics, one of the most reasonable and adopted ways to generalize the concept of temperature in a non-equilibrium state is that of relating it to the Lagrange multipliers associated to the energy constraint.

For the phonon transport in graphene, the approach based on the Lagrange multipliers was followed in [17] (which the interested reader is referred to for the details) within the application of the MEP (see [18,21] for a review of MEP in semiconductors). Let us recall here the main steps.

At equilibrium, the phonon temperatures are related to the corresponding Lagrange multipliers by means of the following:

$$\begin{array}{c} T_{\mu }=\frac{1}{k_{B}\lambda_{W_{\mu }}},\;\;\mu =\mathrm{LO},\mathrm{TO},\mathrm{ZO},K,\mathrm{LA},\mathrm{TA},\mathrm{ZA}. \end{array}$$

If we assume that such relations hold, even out of equilibrium, the definition of a second local temperature can be given in terms of the Lagrangian multipliers as follows.

**Definition** **2.** The local temperature of a system of two or more branches of phonons is $T_{L}^{*}:=\frac{1}{k_{B}\lambda_{W_{L}}}$, where $\lambda_{W_{L}}$ is the common Lagrange multiplier that the occupation numbers of the branches, taken into account, would have if they were in the local thermodynamic equilibrium corresponding to their total energy density, that is, the following:

$$W\left(\lambda_{W_{L}}\right):=\sum_{\mu }W_{\mu }\left(\lambda_{W_{L}}\right)=\sum_{\mu }W_{\mu }\left(\lambda_{W_{\mu }}\right),$$

*where the sum is extended to the considered branches and the functions $W_{\mu }\left(\lambda_{W_{\mu }}\right)$ are found from expressions (13) and (14).*

In other words, we require that $T_{L}^{*}$ is such that, by evaluating all the average phonon energy densities with the Lagrange multiplier given by $1/k_{B}T_{L}^{*}$ and by summing them up, one obtains the value of the total average energy density.

The two definitions of local temperature are equivalent if, and only if, all the relaxation times are equal, that is, the following:

(23) $$\begin{array}{c} \tau_{\mu }=\tau ,\;\mu =\mathrm{LO},\mathrm{TO},\mathrm{ZO},K,\mathrm{LA},\mathrm{TA},\mathrm{ZA}, \end{array}$$

but this assumption is not compatible with the experimental data as clearly indicated in Figure 1, where the relaxation times of all the phonon branches are reported [31]. As a consequence, the two definitions of temperature do not coincide unless all the phonons are in local equilibrium among them, that is, they all have the same local temperature. We notice that $T_{L}^{*}$ is related only to the energy of the system and does not take into account any scattering mechanism. However, the collision terms are now expressed in terms of the Lagrange multipliers associated to the energies. In fact, the strength of the energy production terms is proportional, according to the relaxation times, to the differences between the energy densities relative to the different Lagrange multipliers and $W(\lambda_{W_{L}})$.

$$\begin{array}{c} \frac{W_{\mu }\left(\lambda_{W_{\mu }}\right)-W\left(\lambda_{W_{L}}\right)}{\tau_{\mu }\left(T_{\mu }\right)}. \end{array}$$

## 4. Two Numerical Experiments Investigating the Previous Definitions of the Local Temperature

In this section, we present two numerical experiments to get some insight into the behavior of the phonon system and the phonon temperatures. The first one refers to a suspended graphene sheet subjected to an external electric field with the aim of investigating how the temperatures of the various phonon branches increase due to the interaction with the electrons and among them. In the second one, the electric field is turned off, and we analyze how the phonons go to the equilibrium. Since we are interested in the behavior of the phonon system, we neglect the phonon interaction with the electrons for the sake of simplicity and also because it is weak as confirmed by the significant difference among the phonon temperatures and the electron one when a current flows through the graphene [32]. The influence of the electrons on the final temperature, on the basis of their much smaller surface heat capacity, can be estimated to be around 10%.

### 4.1. First Numerical Experiment

The graphene layer is considered infinite in one dimension, while the other one, which we call longitudinal, is taken as 0.1 μm long. A constant bias is applied to the transversal boundaries. For the simulation, we exploit the macroscopic model for electrons and phonons presented in [17] but using the phonon–phonon relaxation times adopted in [9,10], with $\tau_{K}={\left(1/\tau_{\mathrm{LO}}+1/\tau_{\mathrm{TO}}\right)}^{-1}$ and the temperature $T_{L}$ as the temperature of the local equilibrium distribution functions appearing in the phonon–phonon collision operators; see formula (7). There being no spatial dependence—in this case, $T_{L}$ and $T_{L}^{*}$ only depend on time—one has to solve a system of ordinary differential equations. As said, we focus our attention on the temperatures of the phonons of the various branches and in particular on the temperatures $T_{L}$ and $T_{L}^{*}$.

Unfortunately, a comparison with experiments is not easy because it is not clear what exactly is measured by the instruments.

**Figure 2:**
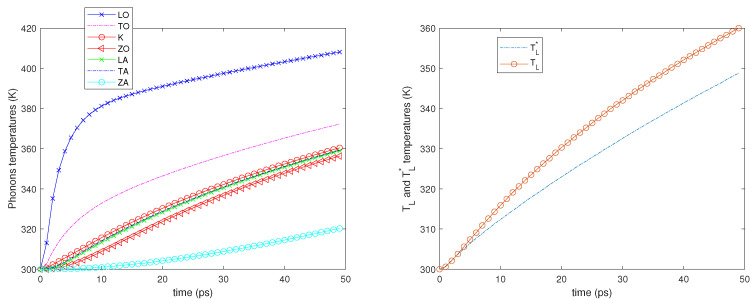
**Left**: The phonon temperatures vs. time. **Right**: $T_{L}^{*}$ and $T_{L}$ vs. time. $\varepsilon_{F}=0.4$ eV, E=2 kV/cm.

**Figure 3:**
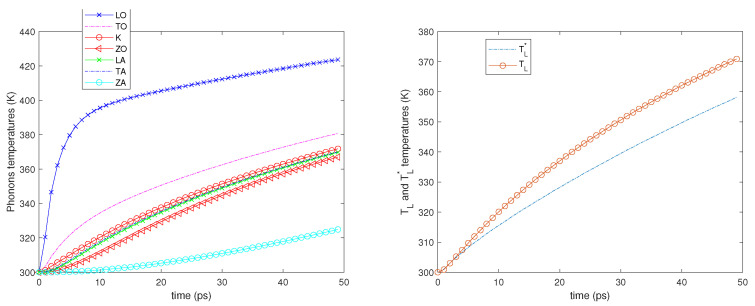
**Left**: The phonon temperatures vs. time. **Right**: $T_{L}^{*}$ and $T_{L}$ vs. time. $\varepsilon_{F}=0.6$ eV, E=2 kV/cm.

**Figure 4:**
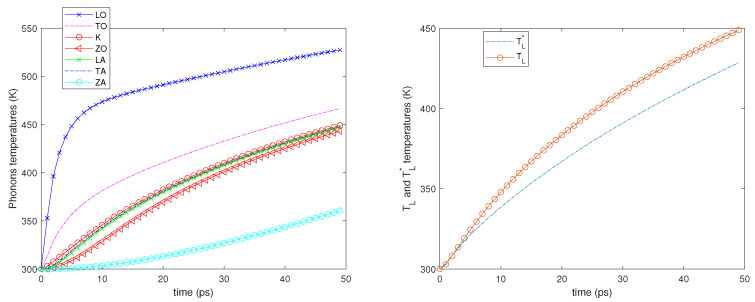
**Left**: The phonon temperatures vs. time. **Right**: $T_{L}^{*}$ and $T_{L}$ vs. time. $\varepsilon_{F}=0.4$ eV, E=5 kV/cm.

**Figure 5:**
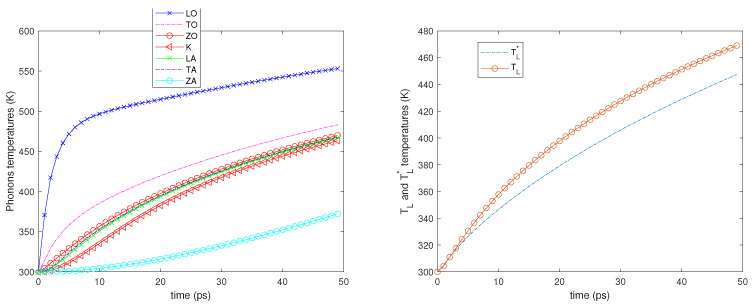
**Left**: The phonon temperatures vs. time. **Right**: $T_{L}^{*}$ and $T_{L}$ vs. time. $\varepsilon_{F}=0.6$ eV, E=5 kV/cm.

Two different values of the electron Fermi energy are considered: $\varepsilon_{F}=$ 0.4 and 0.6 eV, and for each of them two different electric fields are applied: E=2 and 5 kV/cm. A time window of 50 picoseconds is simulated starting from an equilibrium state at room temperature (300 K). In all the cases, see the left-hand sides of Figure 2, Figure 3, Figure 4 and Figure 5; it can be seen that the phonons that have the highest temperature are the LO ones since they have a greater energy exchange with the electrons. The temperature of the ZA phonons is significantly the lowest one, which means that they exchange less energy with the other phonons, while the temperatures of the other branches are near one another.

We also notice that initially, the LO, TO, and K phonon temperatures, in particular that of the LO phonons, increase more rapidly since the phonon–phonon decay acts on a longer time scale. As regards the temperatures $T_{L}$ and $T_{L}^{*}$, see the right-hand sides of Figure 2, Figure 3, Figure 4 and Figure 5; they begin to be substantially different on a time scale comparable with that of the phonon–phonon decay and after some time, their difference remains nearly constant.

### 4.2. Second Numerical Experiment

**Figure 6:**
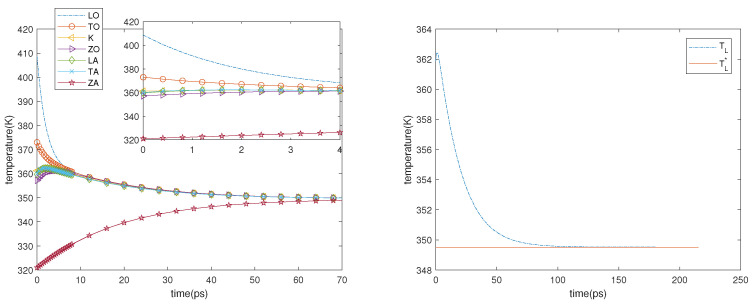
**Left**: Phonon temperatures vs. time in the inset behavior in the first 4 ps is depicted. **Right**: $T_{L}^{*}$ and $T_{L}$ versus time. First case.

**Figure 7:**
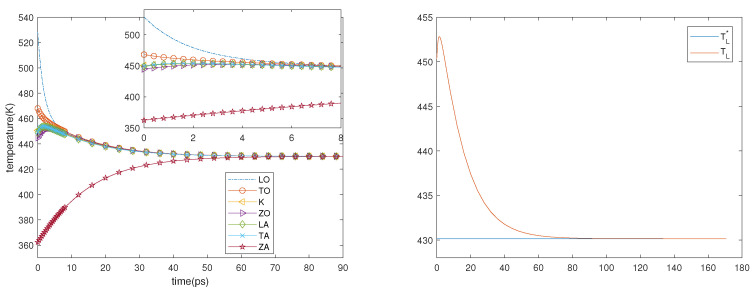
**Left**: Phonon temperatures vs. time, in the inset behavior in the first 8 ps is depicted. **Right**: $T_{L}^{*}$ and $T_{L}$ versus time. Second case.

Guided by the results of the first experiment, we consider a sheet of graphene in which the initial temperatures of the phonons are the final ones of the first experiment. The electric field is turned off and only the phonon–phonon interactions are taken into account; therefore, the physical system can be described by the system of ordinary differential equations, which is obtained from (18) and (19) when no spatial dependence is present. Two cases are considered. In the first one, the initial phonon temperatures are as follows: $T_{\mathrm{LO}}=$ 408.6 K, $T_{TO}=$ 373.0 K, $T_{K}=$ 361.2 K, $T_{ZO}=$ 357.2 K, $T_{LA}=$ 359.7.2 K, $T_{TA}=$ 360.0 K and $T_{ZA}=$ 321.0 K. In the second one, they are as follows: $T_{\mathrm{LO}}=$ 528.4 K, $T_{\mathrm{TO}}=$ 468 K, $T_{K}=$ 450.6 K, $T_{\mathrm{ZO}}=$ 444.8 K, $T_{\mathrm{LA}}=$ 448.7 K, $T_{\mathrm{TA}}=$ 449.2 K, and $T_{\mathrm{ZA}}=$ 362.4.8 K. The first thing that can be seen in the left-hand sides of Figure 6 and Figure 7 is that the ZA phonons (and, though less, also the ZO ones) have a great influence on the final temperature that is reached by the system; moreover, they have a greater inertia. The equilibrium is reached in about 60 ps and the final temperature is $T_{L}^{*}$, which is constant during the process (see the right-hand sides of Figure 6 and Figure 7), in agreement with the energy conservation and with the fact that the right-hand sides of the evolution equations are null if, and only if, all the phonon temperatures are equal to $T_{L}^{*}$.

As regards $T_{L}$, we can see in the right-hand sides of Figure 6 and Figure 7 that after an initial overshoot, which occurs in correspondence with the most rapid variation in the temperatures of the phonons, it decays toward $T_{L}^{*}$. Therefore, the numerical results suggest that $T_{L}^{*}$ has to be intended as the local equilibrium lattice temperature, while the difference between $T_{L}$ and $T_{L}^{*}$ can be interpreted as a measure of how fast the system is trying to approach the equilibrium.

## 5. The Entropy Density of the Phonon System in the Homogeneous Case

The phonon behavior observed in Section 4.2 can be theoretically proved starting from the entropy density of the phonon system, which is given by the following:

$$\begin{array}{c} S=-\frac{k_{B}}{{\left(2\pi \right)}^{2}}\sum_{\mu }\int_{B}\left[g_{\eta }\ln g_{\mu }-\left(1+g_{\mu }\right)\ln\left(1+g_{\mu }\right)\right]dq. \end{array}$$

Substituting in this formula the maximum entropy distributions and neglecting, in agreement with the small anisotropy hypothesis, the quadratic terms in the vector Lagrange multipliers, one obtains the following:

(24) $$\begin{array}{ccc} S & \approx & k_{B}\sum_{\mu }\int \lambda_{W_{\mu }}dW_{\mu }=k_{B}\left\{\sqrt{\frac{\pi }{6\hbar \alpha_{ZA}}}W_{ZA}^{\frac{1}{2}}+\sum_{ac\neq ZA}{\left(\frac{\zeta (3)}{\pi \hbar^{2}c_{ac}^{2}}\right)}^{\frac{1}{3}}\frac{3}{2}W_{ac}^{\frac{2}{3}}\;\right.\\ & + & \left.\sum_{op}\left[\frac{W_{op}}{\epsilon_{op}}\ln\left(1+\frac{A\epsilon_{op}}{{\left(2\pi \right)}^{2}W_{op}}\right)+\frac{A}{{\left(2\pi \right)}^{2}}\ln\left(1+\frac{{\left(2\pi \right)}^{2}W_{op}}{A\epsilon_{op}}\right)\right]\right\}. \end{array}$$

**Remark** **1.** *From the previous expression for each type of phonon, the local temperature can be written as follows:*

(25) $$\frac{1}{k_{B}T_{\mu }}=\lambda_{W_{\mu }}=\frac{\partial S}{\partial W_{\mu }}.$$

*This strongly reminds of the* entropic *definition of temperature:*

$$\frac{\partial S}{\partial u}=\frac{1}{k_{B}T}.$$

*where u is the internal energy and T is the (entropic) temperature of the system. Note that such an approach cannot be used for the whole system because of the lack of a one-to-one correspondence between the total energy and the energies of the single species. Instead, for the whole system, definition 3 can be viewed as a sort of caloric temperature (see [33] for a more complete discussion on the subject).*

The following theorem holds.

**Theorem** **1.** *For any $T\in R^{+}$, the entropy (24) with reverse sign, restricted to the hypersurface $\left\{\left(T_{\mathrm{LO}},T_{\mathrm{TO}},T_{K},T_{\mathrm{ZO}},T_{\mathrm{LA}},T_{\mathrm{TA}},T_{\mathrm{ZA}}\right)\in {\left(R^{+}\right)}^{7}|\sum_{\mu }W_{\mu }\left(T_{\mu }\right)=\sum_{\mu }W_{\mu }\left(T\right)\right\}$, where μ runs over all the phonon types, is a Lyapunov function [34] for the dynamical system as follows:*

$$\begin{array}{ccc} \frac{\partial W_{op}}{\partial t} & = & C_{W_{op}},\;\;op=\mathrm{LO},\mathrm{TO},K,\mathrm{ZO},\\ \frac{\partial W_{ac}}{\partial t} & = & C_{W_{ac}},\;\;ac=\mathrm{LA},\mathrm{TA},\mathrm{ZA}. \end{array}$$

**Proof.**  One has the following:

$$\begin{array}{ccc} -\frac{\partial S}{\partial t} & = & k_{B}\sum_{\mu }\lambda_{W_{\mu }}\left(T_{\mu }\right)\frac{W_{\mu }\left(T_{\mu }\right)-W_{\mu }^{LE}\left(T_{L}\right)}{\tau_{\mu }\left(T_{\mu }\right)}\\ & = & k_{B}\sum_{\mu }\left(\lambda_{W_{\mu }}\left(T_{\mu }\right)-\lambda_{W_{\mu }}\left(T_{L}\right)\right)\frac{W_{\mu }\left(T_{\mu }\right)-W_{\mu }\left(T_{L}\right)}{\tau_{\mu }\left(T_{\mu }\right)}\leq 0, \end{array}$$

where the second equality is due to the energy conservation property of the collision operator (we remind that $\lambda_{W_{\mu }}\left(T_{L}\right)=\frac{1}{k_{B}T_{L}},\;\forall \mu$) and the inequality is due to the fact that the $W_{\mu }$ are increasing functions of their argument, while the $\lambda_{W_{\mu }}$ are decreasing functions. Furthermore, $-\frac{\partial S}{\partial t}=0$ if, and only if, $T_{\mathrm{LO}}=T_{\mathrm{TO}}=T_{K}=T_{\mathrm{ZO}}=T_{\mathrm{LA}}=T_{\mathrm{TA}}=T_{\mathrm{ZA}}$. This implies that any $\left(T,T,T,T,T,T,T\right)\in {\left(R^{+}\right)}^{7}$ is a stable equilibrium state having, as its basin of attraction, the hypersurface $\{\left(T_{\mathrm{LO}},T_{\mathrm{TO}},T_{K},T_{\mathrm{ZO}},T_{\mathrm{LA}},T_{\mathrm{TA}},T_{\mathrm{ZA}}\right)\in {\left(R^{+}\right)}^{7}|\sum_{\mu }W_{\mu }\left(T_{\mu }\right)=\sum_{\mu }W_{\mu }\left(T\right)\}.$ □

**Figure 8:**
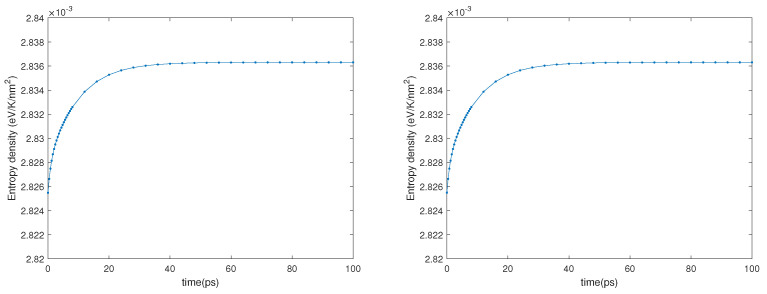
Entropy density vs. time. **Left**: first case of the second experiment. **Right**: second case of the second experiment.

Eventually, in Figure 8, we report the time behavior of the entropy density for the two cases considered in the second numerical experiment, which show that the entropy is a convex function of time.

## 6. Conclusions

Two different definitions of local temperature were compared: one, $T_{L}$, based on the properties of the phonon–phonon collision operator, and the other, $T_{L}^{*}$, based on the energy Lagrange multipliers. Since it is difficult to have a clear meaning of the experimental results, the analysis was based on two numerical experiments represented by simulations of charge and phonon transport in suspended monolayer graphene. The results indicate that the temperature $T_{L}$ has to be intended as a measure of how fast the phonon system is trying to converge to the local equilibrium, while $T_{L}^{*}$ is the local equilibrium lattice temperature. We have also provided an explicit expression of the phonon entropy density in the homogeneous case.

The case of phonon transport in graphene is particularly challenging because, apart from its own interest due to the increasing importance of thermal effects in nano devices, it has several degrees of freedom—the several branches—and each of them possesses a different temperature. Therefore, one is faced with several important questions of non-equilibrium thermodynamics: the definition of temperature, the interaction among the several degrees of freedom, and the identification of a local temperature.

What was found contributes to the complex and controversial debate about the concept of temperature out of equilibrium. The definition based on the collision operator (Definition 1 of the paper) is employed within a kinetic context but it is little explored in a general framework of non-equilibrium thermodynamics; see the quoted review articles [2,3,4] where it is not explicitly mentioned. However, being that this temperature is related to the phonon–phonon interactions, that is, to the anharmonic interaction terms, we can say that it, in some sense, recalls the configurational temperature [6,7].

The definition based on the energy Lagrange multipliers is consistent with the statistical approach and the application of the maximum entropy principle [20]. Moreover, as shown in the present paper, for each species, it is related to the entropic definition of non-equilibrium temperature, while for the whole system, such an approach leads to a sort of caloric definition of local temperature out of equilibrium.

## Data Availability

Not applicable.

## References

[1] Lebon G., Jou D., Casas-Vazquez J. (2008). Understanding Non-equilibrium Thermodynamics.

[2] Casas-Vazquez J., Jou D. (2003). Temperature in non-equilibrium states: A review of open problems and current proposals. Rep. Prog. Phys..

[3] Powles J.G., Rickayzen G., Heyes D.M. (2005). Temperatures: Old, new and middle aged. Mol. Phys..

[4] Puglisi A., Sarracino A., Vulpiani A. (2017). Temperature in and out of equilibrium: A review of concepts, tools and attempts. Phys. Rep..

[5] Mushik W. (2014). Contact temperature and internal variables: A glance back, 20 years later. J. Non Equilib. Thermodyn..

[6] Baranyai A. (2000). On the configurational temperature of simple fluids. J. Chem. Phys..

[7] Rugh H.H. (1997). Dynamical approach to temperature. Phys. Rev. Lett..

[8] Castro Neto A.H., Guinea F., Peres N.M.R., Novoselov K.S., Geim A.K. (2009). The electronic properties of graphene. Rev. Mod. Phys..

[9] Coco M., Romano V. (2018). Assessment of the constant phonon relaxation time approximation in electron-phonon coupling in graphene. J. Comput. Theor. Transp..

[10] Coco M., Romano V. (2018). Simulation of electron-phonon coupling and heating dynamics in suspended monolayer graphene including all the phonon branches. J. Heat Transfer..

[11] Lichtenberger P., Morandi O., Schürrer F. (2011). High-field transport and optical phonon scattering in graphene. Phys. Rev. B.

[12] Coco M., Majorana A., Romano V. (2017). Cross validation of discontinuous Galerkin method and Monte Carlo simulations of charge transport in graphene on substrate. Ric. Math..

[13] Coco M., Majorana A., Mascali G., Romano V., Schrefler B., Onate E., Papadrakakis M. (2015). Comparing kinetic and hydrodynamical models for electron transport in monolayer graphene. Proceedings of the VI International Conference on Computational Methods for Coupled Problems in Science and Engineering, COUPLED PROBLEMS.

[14] Coco M., Majorana A., Nastasi G., Romano V. (2019). High-field mobility in graphene on substrate with a proper inclusion of the Pauli exclusion principle. Atti della Accademia Peloritana dei Pericolanti. Cl. Sci. Fis. Mat. Nat..

[15] Romano V., Majorana A., Coco M. (2015). DSMC method consistent with the Pauli exclusion principle and comparison with deterministic solutions for charge transport in graphene. J. Comput. Phys..

[16] Mascali G. (2015). A hydrodynamic model for silicon semiconductors including crystal heating. Eur. J. Appl. Math..

[17] Mascali G., Romano V. (2017). Charge transport in graphene including thermal effects. SIAM J. Appl. Math..

[18] Camiola V.D., Mascali G., Romano V. (2020). Charge Transport in Low Dimensional Structures: The Maximum Entropy Approach.

[19] Di Stefano V., Muscato O. (2019). Local equilibrium and off-equilibrium phenomena in silicon quantum wires. AAPP.

[20] Jaynes E.T. (1957). Information Theory and Statistical Mechanics. Phys. Rev..

[21] Mascali G., Romano V. (2017). Exploitation of the Maximum Entropy Principle in Mathematical Modeling of Charge Transport in Semiconductors. Entropy.

[22] Barletti L. (2017). Hydrodynamic equations for electrons in graphene obtained from the maximum entropy principle. J. Math. Phys..

[23] Morandi O. (2014). Charge transport and hot-phonon activation in graphene. J. Comput. Theor. Transp..

[24] Müller I., Ruggeri T. (1998). Rational Extended Thermodynamics.

[25] Coco M., Mascali G., Romano V. (2016). Monte Carlo analysis of thermal effects in monolayer graphene. J. Comput. Theor. Transp..

[26] Jou D., Lebon G., Casas-Vazquez J. (2009). Extended Irreversible Thermodynamics.

[27] Coco M., Nastasi G. (2020). Simulation of bipolar charge transport in graphene on h-BN. Compel.

[28] Hao Q., Chen G., Jeng M.S. (2020). Frequency-dependent Monte Carlo simulations of phonon transport in twodimensional porous silicon with aligned pores. J. Appl. Phys..

[29] Peraud J.P.M., Hadjiconstantinou N.G. (2011). Efficient simulation of multidimensional phonon transport using energy-based variance-reduced Monte Carlo formulations. Phys. Rev. B.

[30] Muscato O., Castiglione T., Coco A. (2019). Hydrodynamic modeling of electron transport in. gated silicon nanowires transistors. Atti Acc. Pelor. Pericolanti.

[31] Vallabhaneni A.K., Singh D., Bao H., Murthy J., XiuRuan X. (2016). Reliability of Raman measurements of thermal conductivity of single-layer graphene due to selective electron-phonon coupling: A first-principles study. Phys. Rev. B.

[32] Betz A.C., Jhang S.H., Pallecchi E., Ferreira R., Fève G., Berroir J.M., Plaçais B. (2013). Supercollision cooling in undoped graphene. Nat. Phys..

[33] Jou D., Restuccia L. (2016). Caloric and entropic temperatures in non-equilibrium steady states. Phys. A.

[34] Perko L. (1991). Differential Equations and Dynamical Systems.

